# Combining Affymetrix microarray results

**DOI:** 10.1186/1471-2105-6-57

**Published:** 2005-03-17

**Authors:** John R Stevens, RW Doerge

**Affiliations:** 1 Department of Statistics, Purdue University, 150 N. University Street, West Lafayette, Indiana 47907-2067, USA; 2 Department of Agronomy, Purdue University, Lilly Hall of Sciences, 915 W. State Street, West Lafayette, Indiana 47907-2054, USA

## Abstract

**Background:**

As the use of microarray technology becomes more prevalent it is not unusual to find several laboratories employing the same microarray technology to identify genes related to the same condition in the same species. Although the experimental specifics are similar, typically a different list of statistically significant genes result from each data analysis.

**Results:**

We propose a statistically-based meta-analytic approach to microarray analysis for the purpose of systematically combining results from the different laboratories. This approach provides a more precise view of genes that are significantly related to the condition of interest while simultaneously allowing for differences between laboratories. Of particular interest is the widely used Affymetrix oligonucleotide array, the results of which are naturally suited to a meta-analysis. A simulation model based on the Affymetrix platform is developed to examine the adaptive nature of the meta-analytic approach and to illustrate the usefulness of such an approach in combining microarray results across laboratories. The approach is then applied to real data involving a mouse model for multiple sclerosis.

**Conclusion:**

The quantitative estimates from the meta-analysis model tend to be closer to the "true" degree of differential expression than any single lab. Meta-analytic methods can systematically combine Affymetrix results from different laboratories to gain a clearer understanding of genes' relationships to specific conditions of interest.

## Background

Microarray technology allows simultaneous assessment of transcript abundance for thousands of genes. This exciting research tool permits the identification of genes which are significantly differentially expressed between conditions. With the use of microarrays becoming more commonplace, it is not unusual for several different laboratories to investigate the genetic implications of the same condition(s). Each lab may produce its own list of candidate genes which they believe to be related to the condition of interest. As a result of sound statistical approaches, each lab will also have for each candidate gene some quantitative measure that serves as the basis for the claim of statistical significance.

Of interest in this paper are the methods by which these quantitative measures may be combined across labs to arrive at a more comprehensive understanding of the effects of the different candidate genes. Where the term "analysis" is used to describe the quantitative approaches to draw useful information from raw data, the term "meta-analysis" [1] refers to the approaches used to draw useful information from the results of previous analyses. Meta-analysis has been predominantly used in the medical and social sciences, in situations where several studies may have been conducted to investigate the effect of the same treatment, and the researcher seeks to combine the results of the different studies in a meaningful way in order to arrive at a single estimate of the true effect of the treatment. For the current application, meta-analytic approaches can be employed to combine the results from several different labs without having access to the original raw data that yielded the initial results. Such approaches have particular utility with the results of Affymetrix GeneChip^® ^microarrays and other fabricated arrays, where results are given in a uniform format that readily lends itself to comparison between labs and combination across labs.

A measure of the degree or magnitude of differential expression provides more information regarding a gene's relation to a disease or condition of interest than does a statement regarding its significance or nonsignificance. This information is useful because it allows for greater precision of estimation of the gene's effect with respect to the condition of interest. That is, to arrive at a clearer understanding of a gene's true effect relating to the condition of interest, it is most helpful to have a quantitative measure of the magnitude of differential expression rather than a simple declaration of significance.

Prior applications of meta-analysis to microarray data have either sought to combine P-values or to combine results across platforms (i.e., combining Affymetrix and cDNA array results) [2-6]. Combining only P-values, while useful in obtaining more precise estimates of significance, does not provide information that is easily interpretable by a biologist, may not indicate the direction of significance (e.g., up- or down-regulation), and most importantly, gives no information regarding the *magnitude *of the estimated expression change. Similarly, while a "vote-counting" approach based on P-values [6] addresses differences in lists of significant genes from separate experiments, it gives no information regarding the magnitude of the estimated expression change. While an "integrative correlation" approach [5] will help identify genes with reproducible expression patterns, it also does not provide any information regarding the magnitude of the estimated expression change

Previous attempts to combine results across microarray platforms (i.e., technologies) assume that spot intensities or signal values for a given gene can be directly compared even though they represent different segments of the gene. That is, a spot for a given gene on a cDNA array represents the entire gene, while each spot for the same gene on an Affymetrix array represents a specific small section of the gene. Thus, combining results across technologies using only spot intensities is problematic from a biological perspective because the measurements represent different physical quantities. Even if the average spot intensity on an Affymetrix array is used, it is not certain that this average spot intensity value is at all comparable to the spot intensity value of the gene on a cDNA array.

Moreau et al. [4] report that 'after appropriate filtering, ratio and intensity data from different platforms can be compared and are amenable to' be used in a meta-analysis. However, "filtering", or "averaging out" outliers or non-reproducible spots, requires some subjectivity in the method of choice and may force agreement between platforms where no agreement should exist due to fundamental technological differences. Parmigiani et al. [5] attempt to address this problem of cross-platform consistency by identifying a set of genes whose expression patterns are essentially reproducible across platforms. However, even for these "reproducible" genes, there remains the question of how to systematically combine their corresponding results from the several laboratories to arrive at a single quantitative measure of differential expression. At the very least, if results are to be combined across platforms in a meta-analysis, the use of covariates [7] should be employed to account for the underlying differences between oligonucleotide (e.g., Affymetrix) and cDNA platforms. The focus of the current application is restricted to standard Affymetrix microarray results, and a method to combine results across laboratories is proposed and evaluated.

## Results

### Affymetrix technology

The Affymetrix GeneChip^® ^microarray [8] represents individual genes by 25-mer segments (probes) fixed to the chip, and also makes use of mismatch probes differing at position 13. Each gene on the chip is typically represented by the same number of probe pairs on the chip (usually 14–20), although exceptions exist. It is now possible for some organisms' entire genomes to be represented on a single microarray (e.g., Arabidopsis). Appropriately prepared tissue sample is hybridized to the array and the array is scanned, producing raw data consisting of the intensities of the individual spots on the array. These intensities come in pairs, with PM denoting the intensity of a perfect-match probe and MM denoting the intensity of the corresponding mismatch probe.

### Affymetrix algorithms

Affymetrix has developed statistical algorithms [9] that employ these individual spot intensities for the purpose of estimating the true expression levels of individual genes in single samples. Furthermore, the Affymetrix approach compares gene expression levels in two different tissues (samples or treatment conditions) and reports a "signal log ratio" (SLR) with 95 percent confidence bounds. The signal log ratio is the signed *Iog*_2 _of the signed fold change (FC) familiar to biologists [9]. That is, *FC *= 2^*SLR *^if *SLR *≥ 0 and *FC *= (-1)2^-*SLR *^if *SLR *< 0. The algorithm used by Affymetrix to compute the SLR is based on Tukey's biweight algorithm [10] and for each gene takes a weighted average of the *log*_2 _of the ratio of *PM *- *MM *between treatments or conditions, with weights related to the deviations from the median *log*_2 _ratio for the gene, and with adjustments made when *PM *<*MM*. The resulting weighted average is the SLR.

Between the two conditions of interest, each gene either changes its level of expression or the level remains the same. A declaration of significant differential expression results from sufficient evidence that the gene is not expressed the same in the two conditions (i.e., that the SLR differs significantly from zero). Tukey's biweight algorithm provides an estimate for the variability of the SLR and an approximate distribution for the SLR estimate. The Affymetrix software (Microarray Suite Version 5.0, or MAS 5.0) reports a 95 percent confidence interval for the SLR [11], from which the estimated standard error can be computed. Individual laboratories can use this information to make a declaration of significant differential expression.

It should be noted that this SLR estimate represents a measure of differential expression between two chips (generically referred to as a base sample chip and an experimental sample chip). In practice, of course, it is recommended that experiments involve more than two chips, but the current MAS 5.0 algorithm is designed to represent differential expression only between two chips at a time. Other approaches exist to measure differential expression (dChip [12] and RMA [13], for example), and future work will evaluate their performance in meta-analyses. However, for the purposes of this current work, the focus of differential expression will rely on the SLR estimate of differential expression between two chips, because these are the estimates provided automatically by the commercial MAS 5.0 software.

If we let  denote the estimate of the SLR *θ*_*i*,*k *_for gene *k *in lab *i*, and  be the upper bound for the 95 percent confidence interval for the same, then both  and  are reported by the Affymetrix software. The Affymetrix documentation [9] gives the estimated standard error of  as *s*_*i*,*k *_= ( - ) / *t*_*i*,*k*_(.975), where *t*_*i*,*k*_(.975) is the upper .025 critical value of the *t *distribution with *df*_*i*,*k *_degrees of freedom, where *df*_*i*,*k *_= *max*(0.7(*n*_*i*,*k *_- 1), 1), with *n*_*i*,*k *_representing the number of probe pairs representing gene *k *on each array in study *i*. The estimated variance of the SLR estimate  is *v*_*i*,*k *_= .

Accordingly, lab *i *could then test for significant differential expression of gene *k *(i.e., test the hypothesis : *θ*_*i*,*k *_= 0) by use of the test statistic *A*_*i*,*k *_= /*s*_*i*,*k*_. Under , *A*_*i*,*k *_approximately follows the *t *distribution with *df*_*i*,*k *_degrees of freedom. The significance P-value (*P*_*i*,*k*_) for gene *k *in lab *i *is the value such that |*A*_*i*,*k*_| is the upper *P*_*i*,*k*_/2 critical value of the *t *distribution with *df*_*i*,*k *_degrees of freedom, and  is rejected at the *α*_*i*,*k *_level if *P*_*i*,*k *_<*α*_*i*,*k*_. That is, if *P*_*i*,*k *_is sufficiently small, then lab *i *would declare gene *k *significantly differentially expressed.

### Meta-analysis

The general meta-analytic framework [7] assumes that a measurable relationship exists between certain quantities of interest, and *n *independent studies have been conducted to examine this relationship. In turn, this relationship can be quantified so that each study produces an estimate of the relationship. If the estimates are appropriately standardized, then each study's estimate can be termed an "effect size" estimate. An effect size is essentially a standardized quantitative expression of the relationship of interest. For example, several different laboratories may investigate which of two drugs are better at treating a particular disease. In this case, the relationship of interest is the difference between the drugs' effects. If each laboratory produces an estimate standardized such that estimates from all laboratories address the same quantity and are on the same scale, then these estimates are effect size estimates.

There are three main classes of effect size estimates [14]. The first and perhaps most common is the standardized difference estimate, such as Hedges's *g*, similar to the t-statistic in a two sample study: . The second is the standardized relation estimate, such as the sample correlation coefficient *r*. The third is the measure of significance, such as the P-value from a particular hypothesis test. (Although not an effect size in the traditional sense, the measure of significance approach is mentioned here for the sake of completeness.)

In order to be combined across studies, effect size estimates must address the same measure or quantity, be standardized, and (with the exception of P-values, which are combined differently [15]) include some measure of variability of the effect size estimate [16]. Once each study *i *has provided its effect size estimate  and its measure of variability *v*_*i*_, a meta-analysis can be performed. Three main meta-analytic approaches exist: fixed effects, random effects, and hierarchical Bayes. The first two approaches are summarized here in order of increasing complexity, and the third is the subject of Choi et al. [3] and a future research interest. The three approaches are discussed more fully in Cooper and Hedges [14] and DuMouchel and Normand [17].

#### Fixed effects meta-analysis model

Assume that *n *independent studies have provided effect size estimates  and measures of variability *v*_*i*_, *i *= 1, ..., *n*. The most general meta-analytic approach assumes that



with sampling error *ε*_*i *_~ *N*(0, ). That is, each  is an estimate of a true fixed underlying effect size *θ*_*i*_, and it is assumed that *θ*_1 _= ... = *θ*_*n *_with the common value *θ*. This is referred to as the homogeneity assumption and can be interpreted as assuming that all studies examined and provided estimates of the same parameter *θ*, and any differences between the estimates are attributable to sampling error alone. This common value parameter *θ *is estimated as a weighted average of the effect size estimates:



The weights *w*_*i *_are chosen to minimize the variance of , and this is achieved by , where *v*_*i *_is the estimated variance of . The variance of  is .

The underlying assumption of homogeneity : *θ*_1 _= ... = *θ*_*n *_can be tested by use of the test statistic



Under , *Q *is approximately distributed as . Then this *χ*^2 ^distribution can serve as the basis for an approximate test of homogeneity. If *Q *is larger than the upper *α*_*Q *_critical value of the  distribution,  is rejected at the *α*_*Q *_level. Alternatively, the homogeneity P-value *P*_*Q *_is the value such that *Q *is the upper *P*_*Q *_critical value of the  distribution, and  is rejected at the *α*_*Q *_level if *P*_*Q *_<*α*_*Q*_.

The test of significance : *θ *= 0 can be considered by use of the test statistic . Under , *Z *is distributed as *N*(0, 1), and if |*Z*| is larger than the upper *α*_*Z*_/2 critical value of the *N*(0,1) distribution,  is rejected at the *α*_*Z *_level. Alternatively, the significance P-value *P*_*Z *_is the value such that |*Z*| is the upper *P*_*Z*_/2 critical value of the *N*(0, 1) distribution, and  is rejected at the *α*_*Z *_level if *P*_*Z *_<*α*_*Z*_.

Meta-analysis in the context of a microarray experiment assumes that several laboratories have provided quantitative measurements of differential expression (the effect size) for a number of genes along with variability estimates. For the fixed effects model, the homogeneity assumption () provides that for each laboratory the gene is expressed the same, and differences between laboratories are due to sampling error only. On the other hand, the hypothesis  has the biological interpretation that there is no change in gene expression between the conditions of interest. This test of significance identifies genes that are significantly differentially expressed between the two conditions, using information from multiple laboratories.

#### Random effects meta-analysis model

In practice, the homogeneity assumption (and the resulting fixed effects model) tends to be overly simplistic but is presented in this paper for the sake of completeness. This assumption can be relaxed to make the meta-analysis model more appropriate. The basic random effects model [18] assumes *n *independent studies have provided effect size estimates  and measures of variability *v*_*i*_, *i *= 1, ..., *n*. In addition, the model assumes that



In this framework, *θ *is the population mean effect size, and there are two error components, *δ *and *ε*, corresponding to between-study and within-study variability, respectively. Each study seeks to make statements regarding this quantity *θ*, and so takes a sample of individuals from a certain population in order to study the underlying effect size *θ*. However, due to differences between studies such as time, location, equipment, and other uncontrollable (and possibly unknown) factors, each study will in fact be estimating a slightly different quantity. That is, due to differences between studies, study *i *is estimating *θ*_*i*_, a random effect size from the population of all possible effect sizes. The error component *δ*_*i *_~ *N*(0, Δ^2^) is the random deviation of *θ*_*i *_from *θ *(representing variability between studies). In this basic model, Δ^2 ^represents the random variation between studies. Within study *i*, the actual estimate  will vary from the "true" effect size *θ*_*i *_based on which random sample is selected. That is, replicates within a study will result in slightly different estimates of the effect size due to sampling error. Here, *ε*_*i *_~ *N*(0, ) is sampling error (representing variability within study *i*).

*Q *is calculated as in the fixed effects model. (Note that the fixed effects model  assumes that Δ^2 ^= 0.) The random effects model uses this *Q *value to calculate new weights , where



Then the meta-analysis estimate for the population mean effect size *θ *is



The variance of  is . The test of significance  : *θ *= 0 can be considered by use of the test statistic . Under , *Z*_*w *_is distributed as *N*(0, 1), and the significance P-value is calculated in the same manner as in the fixed effects model.

When the random effects meta-analysis is applied in the context of a microarray experiment, again it is assumed that several laboratories have provided quantitative measurements of differential expression (the effect size) for a given gene along with variability estimates. The random effects model assumes that there is some true degree of differential expression for the gene, and each lab is actually estimating a slightly different true degree of differential expression. That is, each laboratory has a slightly different "true" degree of differential expression. In addition, the estimate from each laboratory varies randomly about its true degree of differential expression due to sampling error. Then Δ^2 ^is a measure of the amount of variation between the laboratories' true degrees of differential expression, and the test of significance is used to identify differentially expressed genes by using information across multiple laboratories.

### Meta-analysis with Affymetrix data

Our motivation for applying meta-analytic techniques to microarray data is threefold. First, standard platforms (e.g., Affymetrix) make combining results across labs straightforward and eliminate the usual criticism of meta-analyses that "apples and oranges" are being mixed [16] because the estimates being combined across labs have each been standardized by the same algorithms [9] in such a way that they are in fact estimates of the same underlying effect. Furthermore, any known differences between laboratories such as sample tissue type can be incorporated into the meta-analysis by use of covariates [7]. Second, combining raw data may provide more information than combining results, but raw data are not always easy to obtain, and it is conceivable that raw data may become unavailable while published (or unpublished) results are available. Third, if it can be shown that meta-analysis produces similar results to the pooling of raw data, then it can be argued that meta-analytic approaches are more efficient in the sense that they only require easily obtainable results rather than the raw data.

The uniformity of chip design and data acquisition from Affymetrix oligonucleotide microarray experiments readily lends itself to a meta-analysis. Given *n *studies examining the differences in gene expression between two treatments (e.g., healthy vs. diseased), a meta-analysis can combine each study's signal log ratio (SLR) estimates in a meaningful way by taking the SLR as the effect size estimate. The SLR satisfies the criteria for an effect size (i.e., comparability of estimates, standardization to the same scale, and availability of a variance estimate). The SLR for a given gene represents the degree of differential expression between two conditions, and is directly comparable between labs since it estimates the same physical quantity. The SLR from Affymetrix is standardized in the sense that a SLR of zero means no differential expression is observed, and the algorithms used to produce the SLR place all SLR estimates on the same scale. Finally, a variance for the SLR estimate is provided by the Affymetrix algorithms [9,10].

A general fixed effects model can be employed to perform a meta-analysis to estimate the true effect size (signal log ratio, SLR) *θ*_*k *_of gene *k*. In addition, the test of homogeneity can be evaluated to determine whether the *n *studies are in fact estimating the same true underlying value of *θ*_*k*_, i.e., whether *θ*_1,*k *_= ... = *θ*_*n*,*k*_. If this homogeneity assumption is found to be reasonable, then a test of significance can be considered to determine whether the true signal log ratio *θ*_*k *_is significantly different from zero (i.e., whether gene *k *is significantly differentially expressed between the two conditions). If the homogeneity assumption is deemed unreasonable, then the random effects model can be employed to account for inter-study variability.

### Simulation example

In order to evaluate the usefulness of this meta-analytic approach, a simulation study was conducted. The purpose of this simulation study was to illustrate how the results of the meta-analysis compare with the actual ("truth") simulation setting. A simple simulation model was developed with the sole purpose of generating "raw" probe-level data with certain genes "known" to be differentially expressed. While this model may not account for all sources of possible variability, it is nonetheless adequate for the purposes of the current work.

#### Simulation model

"Raw" probe-level data were generated from a model assuming that mismatch intensities (MM) are random background noise, which is an underlying assumption of the Affymetrix approach [9]. Our investigation of real data indicated that mismatch intensities appear to follow a long-tailed Gamma distribution. Based on this, a random mismatch intensity is simulated for each probe *l *of each gene *k *such that *MM*_*kl *_~ *Gamma*(*α*, *β*), with mean  and variance [19].

In this simulation, larger values of the shape parameter *α *indicate more signal being detected by mismatch probes, with the peak of the distribution of MM intensities being moved away from zero. Larger values of the scale parameter *β *make high MM intensities less likely by pulling in the tail of the distribution. For the purposes of this simulation, it was assumed that mismatch intensities did not vary across labs or treatments.

Once the background mismatch intensities were obtained, the perfect match (PM) intensities were generated via the model

*Y*_*ijkl *_= *μ *+ *L*_*i *_+ *G*_*k *_+ *P*(*G*)_(*k*)*l *_+ *LG*_*ik *_+ *ρ*_*k*_(*T*_*j *_+ *LT*_*ij *_+ *TG*_*jk *_+ *LTG*_*ijk *_+ *TP*(*G*)_*j*(*k*)*l*_) + *ε*_(*ijk*)*l *_    (7)

where *Y*_*ijkl *_is the *log*_2 _of the *PM *- *MM *difference for probe *l *of gene *k *under treatment *j *in lab *i*. *N *labs were considered with each lab using the same two treatments. The term *ρ*_*k *_~ *Bernoulli*(*p*) is 1 if gene *k *is differentially expressed between conditions *j *= 1 and *j *= 2, and is 0 otherwise. The parameter *p *corresponds to the percentage of genes that are differentially expressed, with higher values resulting in more differentially expressed genes. In this model, *L*_*i *_is the effect of lab *i*, *T*_*j *_is the effect of treatment *j*, *G*_*k *_is the effect of gene *k*, *P*(*G*)_(*k*)*l *_is the effect of probe *l *of gene *k*, *ε*_(*ijk*)*l *_is a random error term, and the other terms are interaction effects. To introduce more between-lab variability, the error variance was allowed to be different in each lab. That is, *ε*_(*ijk*)*l *_~ *N*(0, ) for the error terms in lab *i*. Each term (*X*) in the model is assumed to be a random effect from a *N*(0, ) distribution, except for the constant *μ*, the fixed effect *T*_*j*_, and the *ρ*_*k *_term. The parameters *p*, *μ*, *T*_*j*_, *σ*_1_, ..., *σ*_*N*_, and *σ*_*X *_for *X *= *L*, *G*, *P*(*G*), *LG*, *LT*, *TG*, *LTG*, and *TP*(*G*) can be adjusted to introduce various sources of variability in the "observed" simulated data.

These simulated data can be used to generate "observed" SLR estimates for each gene in each lab. These "observed" SLR estimates can then be combined systematically in a meta-analysis. Note that the "true" SLR value for each gene can be obtained by using the same parameter values as in the simulation model but dropping all lab and error terms. Then the adaptive nature of the meta-analytic approach can be illustrated by comparing the "true" SLR values with the estimates from each lab and from the meta-analysis models.

#### Simulated data

**Figure 1:**
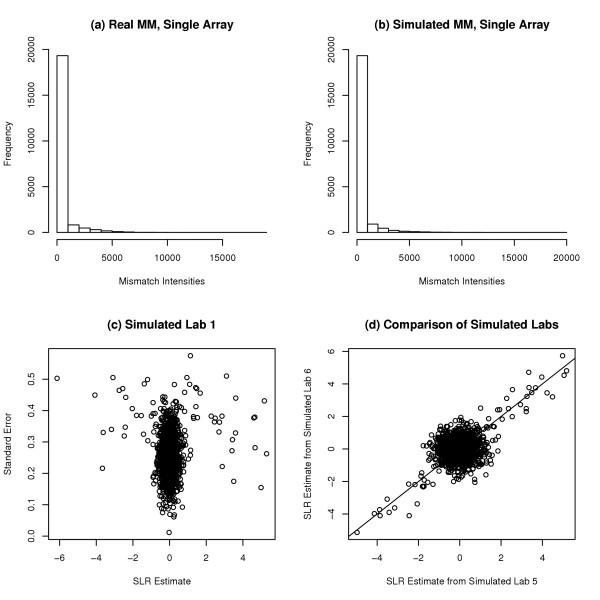
**Summary of real and simulated RN_U34 Affymetrix chip data**. **(a) **The real mismatch (MM) intensities are from a RN_U34 Affymetrix chip, and their histogram closely resembles a Gamma distribution with a long tail. **(b) **The simulated MM intensities are drawn from a Gamma distribution to resemble the real MM intensities. **(c) **The relationship between the SLR from a simulated lab and the standard error of the SLR for each gene on the RN_U34 Affymetrix chip. Large absolute SLR's with small standard errors indicate significant differential expression. **(d) **A comparison of SLR estimates from two simulated labs shows general agreement, with some differences between labs.

The simulation was conducted in the R environment [20] with code requiring the use of the affy package [21] from the Bioconductor project [22,23]. While not the purpose of this investigation, the simulation was performed based upon the Affymetrix rat neuro chip RN_U34 with model parameter settings *N *= 6, *α *= 0.1, *β *= 0.0003, *p *= 0.05, *μ *= 2.5, *σ*_*L *_= 0, *σ*_*G *_= 0.5, *σ*_*P*(*G*) _= 0.3, *σ*_*LG *_= 0.1, *T*_1 _= -0.2, *T*_2 _= 0.2, *σ*_*LT *_= 0.1, *σ*_*TG *_= 1.0, *σ*_*LTG *_= 0.13, *σ*_*TP*(*G*) _= 0.5, *σ*_1 _= .48, *σ*_2 _= .60, *σ*_3 _= .72, *σ*_4 _= .84, *σ*_5 _= .96, and *σ*_6 _= 1.08. These parameter settings were selected to produce a distribution of MM intensities similar to that observed in real data (Figure 1a,b) and to force the distribution of signal log ratio (SLR) estimates to fall within a reasonable range with some variation between laboratories (Figure 1c,d).

Most SLR estimates were near zero (Figure 1c), indicating nondifferential expression, while some genes had larger absolute SLR's with smaller standard errors, an indication of significant differential expression. The data were simulated such that there were similar patterns between labs while allowing for lab differences, as evidenced by a comparison of the SLR's from two simulated labs (Figure 1d). While the estimates from the two simulated labs were clearly similar, there were obvious differences between the labs, although not as different as could be observed in real data. As a result, these two labs might produce slightly different lists of significantly differentially expressed genes. The simulation parameters can be adjusted to introduce varying degrees of difference between experiments, and this will affect the final claim made by the meta-analysis regarding statistical significance of differential expression.

#### Fixed effects meta-analysis results

**Figure 2:**
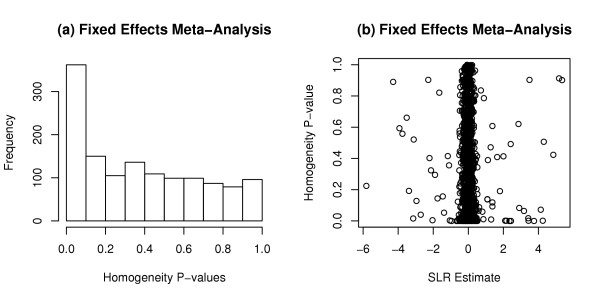
**Fixed effects meta-analysis of simulated results**. (a) The abundance of smaller homogeneity P-values indicates widespread violation of the homogeneity assumption. (b) The plot of homogeneity P-values versus fixed effects SLR estimates shows that this lack of homogeneity exists across a large range of SLR estimates.

The results from the six simulated labs were combined using the fixed effects meta-analysis model (Figure 2). The test of homogeneity : *θ*_1,*k *_= ... = *θ*_6,*k *_was performed for each gene *k*, *k *= 1, ..., 1322 (the RN_U34 chip has 1322 features or reference sequences), and the P-values for each  were summarized in a histogram of homogeneity P-values (Figure 2a). Clearly there was widespread violation of the homogeneity assumption, as evidenced by the abundance of smaller homogeneity P-values.

When the False Discovery Rate (FDR) [24] was controlled at 0.05, 88 of the 1322 genes failed the homogeneity test. That is, there appeared to be significant interlaboratory differences, such that the laboratories did not appear to provide estimates of the same true degree of differential expression for all genes. This appeared to be true for genes across a wide range of fixed effects meta-analysis SLR estimates, as evidenced by the lack of a clear relationship between fixed-effects SLR estimates and homogeneity P-values (Figure 2b). As a result, the random effects meta-analysis model was deemed more appropriate to adjust for the lack of homogeneity.

#### Random effects meta-analysis results

**Figure 3:**
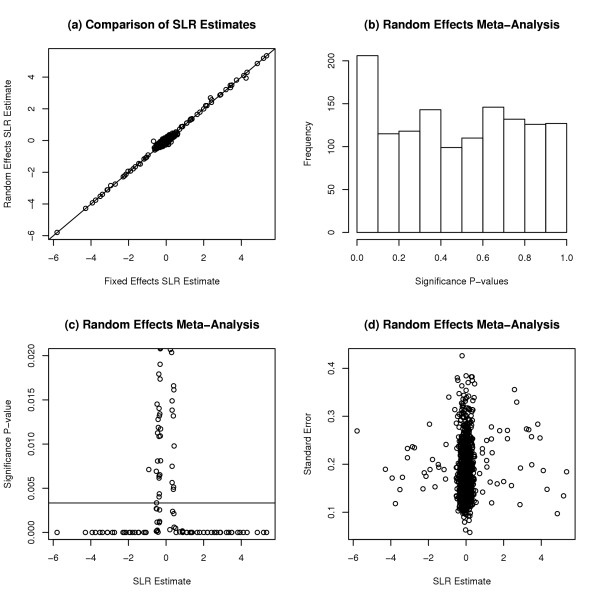
**Random effects meta-analysis of simulated results**. **(a) **A comparison of the fixed effects and random effects meta-analysis estimates of SLR shows general agreement between the models. **(b) **The histogram of significance P-values shows an abundance of significantly differentially expressed genes, as evidenced by the large number of smaller significance P-values. **(c) **The smallest significance P-values tended to occur for genes whose random effects meta-analysis SLR estimates were large in absolute value. The reference line is the P-value cut-off used to control the False Discovery Rate at 0.05. Using this cut-off, the random effects meta-analysis declared 72 of the 1322 genes significantly differentially expressed. **(d) **Demonstration of how the SLR's estimated from the meta-analysis relate to their standard errors.

The same data from the six simulated labs were used in a random effects meta-analysis, and the resulting SLR estimates were similar to those from the fixed effects meta-analysis (Figure 3a). The test of significance : *θ*_*k *_= 0 was performed for *k *= 1, ..., 1322 (i.e., for all 1322 genes), and the P-values for each value of *k *were summarized in a histogram of significance P-values (Figure 3b). As a result of the parameter selections for the simulation, an abundance of small P-values was observed, indicating a large number of significantly differentially expressed genes. A comparison of the meta-analysis SLR estimates with the significance P-values (Figure 3c) showed a trend of smaller P-values for larger absolute SLR. Similar to the results from a single lab (Figure 1c), most meta-analysis SLR estimates were close to zero (Figure 3d), but the standard errors were slightly lower overall for the meta-analysis estimates, after combining the SLR estimates across labs.

**Figure 4:**
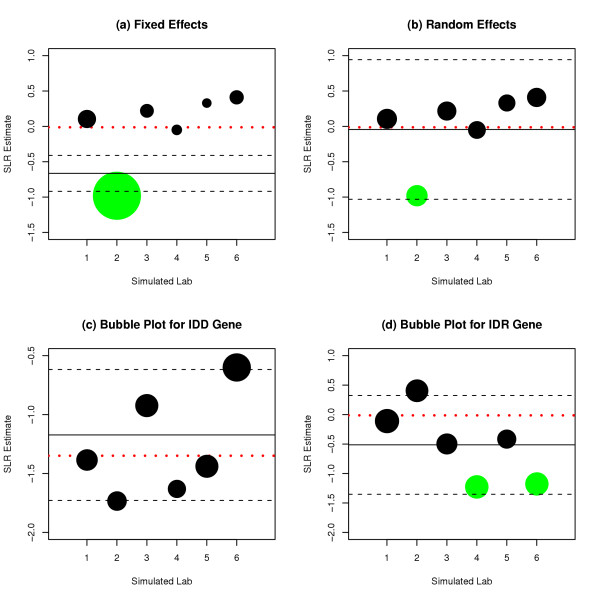
**Bubble plots from the simulation example**. Bubble area is proportional to weights used in the meta-analysis. Dashed lines represent the 95 percent confidence interval for the true value of the SLR, adjusted to control the FDR for all 1322 genes at 0.05. The red dotted line represents the true SLR value. The green bubbles represent labs which claimed significant differential expression for the gene. When zero lies outside the confidence interval, the meta-analysis declares the gene significantly differentially expressed. **(a) **SLR estimates from the six labs for a particular gene, with fixed effects weights. **(b) **Plot for the same gene as in (a), but with random effects weights. **(c) **Plot for one of the twenty-one Integration-Driven Discovery (IDD) genes declared significant by none of the six simulated labs but significant by the random effects meta-analysis. **(d) **Plot for one of the four Integration-Driven Revision (IDR) genes declared significant by multiple labs but not significant by the random effects meta-analysis.

The differences between the fixed effects and random effects models can be summarized by considering bubble plots for a single gene (Figure 4a,b), with bubble area proportional to weights used in the meta-analysis. For this particular gene, laboratory 2 estimated a SLR considerably smaller than the SLR's from the other labs with very small variance and hence very large weight in the fixed effects meta-analysis. As a result, the fixed effects meta-analysis estimated the true SLR for this gene to be closest to the SLR from lab 2. Such a result would call into question the SLR estimate from lab 2 for this gene. The random effects model took this lack of homogeneity into account and appropriately down-weighted the SLR estimates for this gene from all six labs. In particular, the weight for this gene in lab 2 was reduced from 88.5 in the fixed effects model to 1.8 in the random effects model. For comparison, the weights for this gene in the other five labs ranged from 3.0 to 11.9 in the fixed effects model and from 1.2 to 1.6 in the random effects model. Whereas the fixed effects model declared this gene significantly differentially expressed, the random effects model did not (controlling the FDR at 0.05 in both models). Thus, the random effects model was not overly influenced by any single lab's SLR estimate.

#### Comparing simulated results and "truth"

When the FDR was controlled at 0.05, 72 of the 1322 genes were declared by the random effects meta-analysis to be significantly differentially expressed based on the results of the test of significance . Individually, the six labs identified between 44 and 58 significantly differentially expressed genes (controlling the FDR at 0.05 for each lab) (Table 1). For each lab, most of its significant genes were declared significant by both the fixed effects and random effects meta-analyses.

These results demonstrate how a meta-analysis handles discrepancies between labs. A meta-analysis can be useful in finding genes that are statistically significantly differentially expressed and not just declared significant by one or more labs due to random variation between labs. For example, lab 1 declared 46 genes significant and lab 2 declared 49 genes significant, but these two labs declared only 33 of the same genes significant (Table 1). These 33 are not necessarily the most significant in either lab. That is, the 33 are not necessarily the genes with the smallest lab 1 P-values or smallest lab 2 P-values, but are those genes with the smallest P-values from both labs. Alternatively, rather than considering all genes declared significant by any of the labs, the random effects meta-analysis combines information across all six labs in a well-structured manner and declares 72 genes significantly differentially expressed.

**Figure 5:**
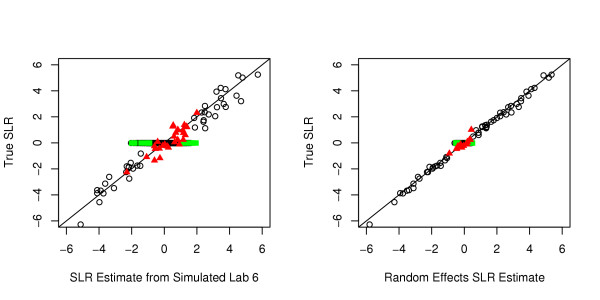
**Comparison of SLR estimates with true values from simulation example**. Green squares represent type I errors, genes incorrectly claimed as differentially expressed. Red triangles represent type II errors, genes incorrectly claimed to be not significantly differentially expressed. The SLR estimates from the random effects meta-analysis tend to approximate the true values much better than does any single lab.

While the numbers of correctly identified differentially expressed genes do not vary drastically between the individual labs (Table 1), the meta-analyses tend to correctly identify a higher number of differentially expressed genes. A comparison of the results from this SLR-based meta-analysis with the results from a previously-proposed meta-analysis approach based on combining P-values [2] is also summarized in Table 1. A slight modification to this P-value approach was necessary to account for differences in experimental design. Where the previous approach implicitly required multiple control and experimental sample arrays from each lab, this simulation data (as well as the real data presented subsequently in this work) did not satisfy this requirement in all labs. To modify the previous approach for the current data, probe-specific perfect match (PM) intensity differences between the experimental and control conditions were used to obtain a paired t statistic. Then the same permutation approach [2] was used to obtain a significance P-value for each gene in each laboratory based on this paired t statistic, and these P-values were combined across laboratories as previously proposed. In general, the SLR-based approach presented here tended to result in more genes found significantly differentially expressed by the meta-analysis than this previously proposed P-value approach (Table 1). In addition, the P-value approach does not provide a final quantitative estimate of the degree of differential expression for each gene, as does the currently proposed SLR-based approach. The meta-analysis SLR estimates tend to be much closer to the true SLR values than do the estimates from individual labs (Figure 5).

#### Integration-Driven Discovery (IDD)

One of the benefits of a meta-analysis is also one of the benefits of pooling raw data, that is the increased power to detect significant differences. It is possible that while a given gene is not declared significantly differentially expressed by any one lab, the combination of results across labs in a meta-analysis provides sufficient evidence to declare significant differential expression. Choi et al. [3] use the term "Integration-Driven Discovery" (IDD) to refer to a gene identified as differentially expressed by the results of a meta-analysis, but not identified as differentially expressed by any of the individual studies or labs. In this case, the term "integration" is used in the unification sense rather than the mathematical, since the results of several different studies are being integrated into a single meta-analysis.

As shown in Table 2, our particular simulation study produced 21 IDD's (i.e., 21 of the 72 genes declared significant by the random effects meta-analysis were not declared significant by any of the six labs). Of these 21 IDD's, 6 were truly differentially expressed; that is, our simulation study produced 6 true IDD's and 15 false IDD's. An examination of the SLR estimates for these IDD genes (Figure 4c) indicated that IDD's will tend to occur when 'small but consistent' [3] effect size estimates are combined. In addition, high variability of each lab's estimate may cause individual labs to not declare a gene significant while the meta-analysis estimate will have a lower variance, making a declaration of significance more likely.

#### Integration-Driven Revision (IDR)

In the simulation results presented here, there were 4 genes declared significant by at least two of the simulated labs that were not declared significant by the random effects meta-analysis (Table 2). A closer examination of the SLR estimates for these particular genes (Figure 4d) revealed that while at least two of the labs individually declared the gene significant, the SLR estimates between the six labs differed sufficiently to make the variance of the meta-analysis SLR estimate large. This increased variance of the meta-analysis SLR estimate caused the meta-analysis to declare these genes not significant. In addition, some labs' variability estimates may be artificially low due to chance, thus forcing a false declaration of differential expression at the individual lab level. The random effects meta-analysis is able to account for this possibility by down-weighting overly influential results.

We introduce the term "Integration-Driven Revision" (IDR) to describe a gene identified as differentially expressed by multiple studies or labs, but determined by the results of a meta-analysis to be not differentially expressed. While multiple laboratories might promote such a gene for further study because of its large and significant effect size, the meta-analysis would conclude that, due to the inconsistencies in effect sizes across labs, the gene is not significantly differentially expressed. Whereas Integration-Driven Discoveries (IDD's) will tend to occur when 'small but consistent' [3] effect size estimates are combined, Integration-Driven Revisions (IDR's) will tend to occur when large but inconsistent effect size estimates are combined. Of the 4 IDR's made in this simulated study, 3 were not truly differentially expressed; that is, our simulation study made 3 true IDR's and 1 false IDR's.

As noted previously, the simulation parameters can be adjusted to introduce varying degrees of difference between experiments. Increased inter-laboratory variability, or greater inconsistency among effect size estimates, will tend to affect the numbers of IDD's and IDR's made by the meta-analysis. Because IDD's occur when effect size estimates are small but consistent and IDR's occur when effect size estimates are large but inconsistent, greater inter-laboratory variability will tend to result in fewer IDD's and more IDR's being made.

### Real data example

Several laboratories have investigated the genetic basis for EAE (experimental autoimmune encephalomyelitis, the mouse model for human multiple sclerosis) by use of Affymetrix technology, and have reported their findings in published papers [25-29]. In each of these published papers, mention is made of appropriate care of the mice following the ethics guidelines at the respective institutions. The three laboratories providing data are Offner [26,29], Carmody [28], and Ibrahim [25,27]. Each laboratory measured gene expression in a base (naive or control) sample and in an experimental (EAE-induced) sample, with some laboratories using multiple experiments. For the current purposes, an "experiment" is a single array-to-array comparison to study differential expression. Seven total experiments from the three laboratories are summarized in Table 3. While not all labs used the same measure of differential expression in their publications, here the Affymetrix MAS 5.0 algorithm [11] provides the SLR estimates for each lab from the respective raw data sets.

The use of different Affymetrix chip versions presents a non-trivial challenge in comparing and combining results across laboratories. The same gene may be represented on two different chip versions, and yet the names reported by the two chips may differ. Also, different sets of probes may represent the same gene on different chip versions, resulting in different probe set names on different chip versions. For example, the gene *1200011I18Rik *on chip Mu11KsubA is identified by Probe Set ID AA000151_at, while on chip MG_U74Av2 it is Probe Set ID 104759_at. Furthermore, different chip versions may have different sets of genes represented on them. In order to combine the results across labs (and consequently, across chip versions), each gene must have a "name" recognized by all chips in the meta-analysis.

Previous meta-analyses of microarray results ([2,6], for example) have relied on Unigene cluster numbers to essentially achieve a uniform gene naming scheme across chip versions and platform types. Other recent work [30] proposed combining raw data from common probes into new probesets based on Unigene clusters. Because the focus of the current work is on combining the results of the Affymetrix algorithms, SLR estimates corresponding to the same Unigene cluster numbers are combined across all experiments. This approach will allow a gene to have multiple SLR estimates (corresponding to different original probe set names) from the same experiment. The Unigene number corresponding to each probe set on an array is available through the NetAffx feature [31] of the Affymetrix website [8].

**Figure 6:**
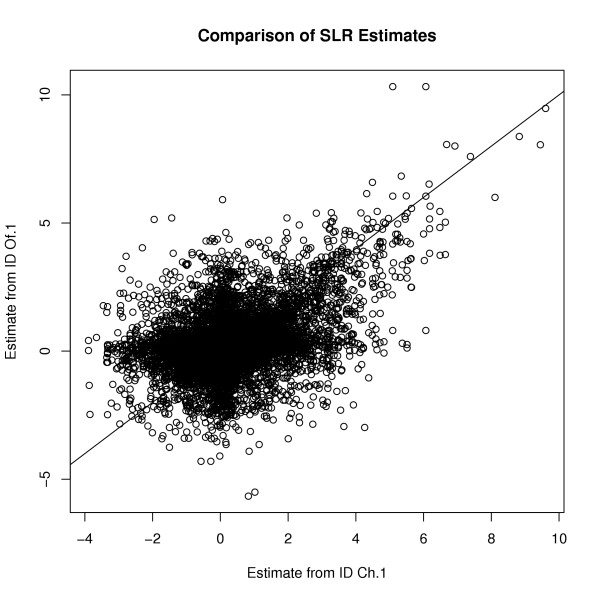
**Comparison of SLR estimates from two experiments in the EAE example**. This plot illustrates the variation between experiments and the consequent need for a method of systematically combining results from different experiments.

There were considerable differences in the SLR estimates from the different labs, as represented in Figure 6. Some of these differences may be due to the use of different mouse strains, tissue types, and chip versions in the different laboratories. However, even experiments from the same laboratory tended to show disagreement, highlighting the need for biological replicates to provide more precise estimates of the degree of differential expression for each gene. This inter-laboratory variability also illustrates the need for methods to systematically combine results across laboratories. The fixed effects and random effects meta-analysis models were employed to combine these estimates across all laboratories for each Unigene number.

Similar to Table 1 for the simulated example, Table 4 summarizes the overlap in the numbers of genes declared significantly differentially expressed by each experiment in this observed data example. Based on the results of the random effects model, 3,671 genes are identified as statistically significantly differentially expressed. There were 12,775 unique Unigene numbers represented by the genes across all arrays in this meta-analysis, so approximately 28.7% of the genes represented were declared significantly differentially expressed. This may support the prediction made by Carmody et al. [28] that about 28.9% of the genes in the mouse genome 'may be relevant for autoimmune inflammation', or affected by EAE. Similar to the simulated data (Table 1), a comparison of the results from this SLR-based meta-analysis with the results from an implementation of the previously proposed P-value approach [2] indicates that even with real data, the SLR-based approach tends to identify more genes as significantly differentially expressed (Table 4).

**Figure 7:**
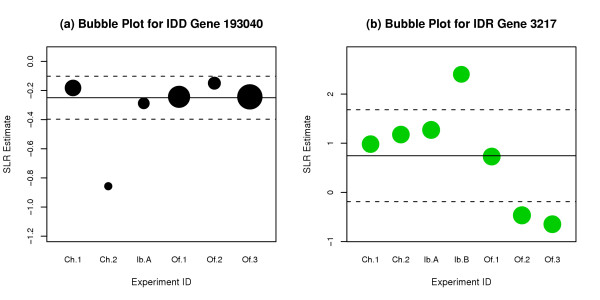
**Bubble plots from the EAE example**. Bubble area is proportional to weights used in the meta-analysis. Dashed lines represent the 95 percent confidence interval for the true value of the SLR, adjusted to control the FDR for all 1322 genes at 0.05. The green bubbles represent experiments which claimed significant differential expression for the gene. When zero lies outside the confidence interval, the meta-analysis declares the gene significantly differentially expressed. **(a) **Bubble plot for one of the sixty-five Integration-Driven Discovery (IDD) genes declared significant by none of the seven observed experiments but significant by the random effects meta-analysis. **(b) **Bubble plot for one of the thirty-two Integration-Driven Revision (IDR) genes declared significant by all seven observed experiments but not significant by the random effects meta-analysis.

As in the simulated data, the meta-analysis of these observed data produced Integration-Driven Discoveries (IDD's) and Integration-Driven Revision (IDR's). Similar to Table 2 for the simulated data, Table 5 summarizes these findings for the observed data. There were 65 IDD's and 5518 IDR's made. Of the IDR's, 32 were made for genes declared significantly differentially expressed by all seven experiments but not by the random effects meta-analysis. Figure 7 presents bubble plots for representative IDD and IDR genes from the observed data, similar to Figure 4 for the simulated data. As in the simulated data, IDD's tend to occur when small but consistent effect sizes are combined, and IDR's tend to occur when large but inconsistent effect sizes are combined. (See Additional file 1 : EAE.Random.Effects.Results.csv for the final estimates for all 12,775 genes.)

## Discussion

Before any clinical decision is made based on the results of a meta-analysis, a biological validation of the results should be performed. Microarray technology is well-suited for hypothesis generation, and a meta-analysis can be used to effectively combine results across multiple laboratories to refine the list of candidate genes deserving biological validation. This approach will tend to yield more informative results when each lab has used biological and technical replicates in their experimental design [32]. The use of replicates at the laboratory level provides both added power to detect differential expression and more precise estimation of the true degree of differential expression for each gene under consideration.

The model used to generate data for the simulation example can be adjusted to account for various sources of variation and relationships between genes. It is of great interest to investigate how such relationships affect the outcome of a meta-analytic approach. An extension of the fixed effects and random effects models to the hierarchical Bayes approach is also being investigated with the hope of improving the meta-analysis approach as applied to microarrays and to incorporate prior knowledge. Included in this extension is the use of covariates in the meta-analysis framework to account for known differences between labs and the appropriate modeling of possible dependence among effect size estimates. We feel the use of covariates will provide insight into the effects of different labs, tissues, and microarray platforms on the observed differential expressions of genes. Separating out the effects of these covariates will facilitate the identification of those genes which are differentially expressed between two conditions rather than appearing differentially expressed due to external influences such as lab, tissue, or platform. For example, the differences observed between experiments from the same laboratory (Table 4) may be explained by differences in mouse strain or tissue sample, and the inclusion of covariate information in the model would adjust for this.

In the examples presented here, all studies involved used the Affymetrix platform and the data were summarized using the same normalization strategy with the MAS 5.0 algorithm [11]. When multiple studies have employed different platforms (such as cDNA and other oligonucleotide arrays) or normalization strategies, then some adjustments to the approach presented here will be necessary. In particular, a readily-available quantitative measure of differential expression common to all platforms involved is needed. In addition, it will be of great interest to consider the effect of a platform covariate in the extended meta-analysis model.

Although we have demonstrated our approach using both simulated rat data and real observed data from essentially genetically homogeneous mice, its utility with human data is of great interest. Along with the increased variability in human data comes an increase in the information about each individual subject and subpopulation. Therefore the incorporation of such covariate information is an important subject of our future work. We anticipate that the use of covariate information with human data will be particularly informative in identifying biologically significant subpopulations - for example, in identifying genes that are related to a disease in one subpopulation but not in another.

## Conclusion

The signal log ratio (SLR), automatically reported by MAS 5.0 [11], is naturally suited to serve as an effect size estimate in a meta-analysis of results from multiple laboratories. In order to perform a meta-analysis of microarray results as presented here, the following components are needed for each probe set from each experiment: the corresponding Unigene ID, the SLR estimate, and the estimated variance of the SLR estimate. The random effects meta-analysis model is better suited than the fixed effects model for the analysis of microarray results because of the lack of homogeneity of effects from different laboratories. Genes not declared significantly differentially expressed by any single lab but then declared significantly differentially expressed by the meta-analysis are referred to as Integration-Driven Discoveries, or IDD's [3]. In addition to the identification of IDD's, our meta-analysis method identified genes declared significantly differentially expressed by multiple (and possibly all) laboratories but not significantly differentially expressed by the meta-analysis. These genes are referred to as Integration-Driven Revisions, or IDR's. The simulation example demonstrated how the final SLR estimates from the meta-analysis models tend to be much closer to the "true" SLR values than do the SLR estimates from any single lab. These meta-analytic approaches to microarray results provide a systematic method to combine results from different laboratories with the purpose of gaining clearer insight into the true degree of differential expression for each gene.

## Authors' contributions

For this research RWD initiated the underlying concept of meta-analysis as applied to microarray technology, and coordinated the focus. RWD is the Ph.D. advisor of JRS. JRS developed and evaluated the simulation model, wrote the R code for the respective analyses and graphical displays, and drafted the manuscript. Both authors read and approved the final manuscript.

## Supplementary Material

Additional File 1 **EAE.Random.Effects.Results.csv **Summary of random effects meta-analysis model results for all 12,775 genes, including SLR estimates, declaration of statistically significant differential expression, and status as an Integration-Driven Discovery (IDD) or Integration-Driven Revision (IDR). Note that the Gene column refers to Unigene cluster number unless the entry has an extension such as _at, in which case it refers to an Affymetrix probe set name for which no Unigene number was available. This file is comma-delimited and can be opened in Microsoft Excel. Click here for file

## Figures and Tables

**Table 1:** Comparison of results from the simulated data. Comparison of numbers of genes in common declared significant (i.e., significantly differentially expressed) by simulated labs 1 through 6, the SLR-based fixed effexts and random effects meta-analyses, and the previously proposed P-value-based meta-analysis [2]. The (*i*, *j*)^*th *^element of this table is the number of genes declared significant by both lab *i *and lab *j*, with F here representing the fixed effects meta-analysis, R the random effects meta-analysis, and T the "truth" behind the simulation model. P represents the meta-analysis based on P-values [2]. Each lab (and meta-analysis) had the False Discovery Rate (FDR) controlled at 0.05.

	Simulated Lab						Meta-Analysis			
								
	1	2	3	4	5	6	Fixed	Random	P-value	Truth
	
1	46	33	34	34	31	31	43	35	33	35
2		49	34	37	31	34	47	39	35	38
3			54	34	32	36	44	41	38	41
4				51	30	35	48	38	35	39
5					44	32	39	37	34	37
6						58	48	40	37	41
F							137	72	45	58
R								72	45	56
P									45	45
T										70

**Table 2:** Summary of results for the simulated data. Numbers of genes declared significant (i.e., significantly differentially expressed) by different numbers of labs and the fixed and random effects meta-analyses in the simulation example. The False Discovery Rate (FDR) for the meta-analyses and each lab separately was controlled at 0.05. There were **21 **Integration-Driven Discoveries (IDD's) and **4 **Integration-Driven Revisions (IDR's).

	Fixed Effects Model		Random Effects Model	
	
Num. of Labs Declaring	Number of Genes Declared		Number of Genes Declared	
Significance	Not Significant	Significant	Not Significant	Significant
0	1152	51	1182	**21**
1	33	38	64	7
2	3	8	**4**	4
3	0	4	0	4
4	0	3	0	3
5	0	7	0	7
6	0	26	0	26

	1188	137	1250	72

**Table 3:** Summary of observed data for the EAE example. A summary of the seven observed experiments involving EAE data to be combined in a meta-analysis.

Lab	Experiment ID	Base Sample	Experimental Sample	Chip Version
Offner	Of.1	Of 1. Naive	Of1.EAE1	MG_U74Av2
Offner	Of.2	Of2.Naivel	Of2.EAEl	MG_U74Av2
Offner	Of.3	Of2.Naive2	Of2.EAE2	MG_U74Av2
Carmody	Ca.1	Ca.Naive1	Ca.Acute1	MG_U74A
Carmody	Ca.2	Ca.Naive2	Ca.Acute2	MG_U74A
Ibrahim	Ib.A	Ib.ControlA	Ib.PeakA	Mu11KsubA
Ibrahim	Ib.B	Ib.ControlB	Ib.PeakB	Mu11KsubB

**Table 4:** Comparison of results from the EAE example. Comparison of numbers of genes in common declared significant (i.e., significantly differentially expressed) by the observed experiments, the SLR-based fixed effects and random effects meta-analyses, and the previously proposed P-value-based meta-analysis [2]. Each experiment (and meta-analysis) had the False Discovery Rate (FDR) controlled at 0.05.

	Observed Experiment ID							Meta-Analysis		
								
	Ib.A	Ib.B	Ca.1	Ca.2	Of.1	Of.2	Of.3	Fixed	Random	P-value
	
Ib.A	2952	402	1327	1253	1474	1354	1349	2336	1216	265
Ib.B		2902	996	950	1093	1067	1065	2456	1546	236
Ca.1			4471	2834	2797	2476	2461	3548	1555	402
Ca.2				4165	2646	2301	2324	3243	1464	333
Of.1					5001	2763	2807	3834	1578	355
Of.2						4911	3035	3669	1344	335
Of.3							5041	3728	1289	305
F								8263	3623	388
R									3671	205
P										453

**Table 5:** Summary of results from the EAE example. Numbers of genes declared significantly differentially expressed by different numbers of experiments and the fixed and random effects meta-analyses in the observed data example. The False Discovery Rate (FDR) for the meta-analyses and each experiment separately was controlled at 0.05. There were **65 **Integration-Driven Discoveries (IDD's) and 5518 Integration-Driven Revisions (IDR's). There were **32 **IDR's that had been declared significantly differentially expressed by all seven experiments.

	Fixed Effects Model		Random Effects Model	
	
Number of Experiments Declaring	Number of Genes Declared		Number of Genes Declared	
Significance	Not Significant	Significant	Not Significant	Significant
0	1792	301	2028	**65**
1	804	2265	1558	1511
2	749	1409	1869	289
3	625	1512	1662	475
4	328	1319	1084	563
5	152	908	617	443
6	54	464	254	264
7	8	85	**32**	61

	4512	8263	9104	3671
